# Association between *ABHD1* and *DOK6* polymorphisms and susceptibility to Hirschsprung disease in Southern Chinese children

**DOI:** 10.1111/jcmm.16905

**Published:** 2021-09-20

**Authors:** Chaoting Lan, Yuxin Wu, Ning Wang, Yun Luo, Jinglu Zhao, Yi Zheng, Yan Zhang, Lihua Huang, Yun Zhu, Lifeng Lu, Wei Zhong, Jixiao Zeng, Huimin Xia

**Affiliations:** ^1^ The First Affiliated Hospital of Jinan University Guangzhou China; ^2^ Guangzhou Medical University Guangzhou Guangdong China; ^3^ Department of Neonatology Guangzhou Baiyunshan Hospital Guangzhou Guangdong China; ^4^ Department of Pediatric Surgery Guangzhou Institute of Pediatrics Guangzhou Women and Children’s Medical Center Guangzhou Medical University Guangzhou Guangdong China

**Keywords:** ABHD1, association, DOK6, Hirschsprung disease

## Abstract

Hirschsprung disease (HSCR) is an infrequent congenital intestinal dysplasia. The known genetic variations are unable to fully explain the pathogenesis of HSCR. The α/β‐hydratase domain 1 *(ABHD1)* interferes with the proliferation and migration of intestinal stem cells. Docking protein 6 (*DOK6*) is involved in neurodevelopment through *RET* signalling pathway. We examined the association of *ABHD1* and *DOK6* genetic variants with HSCR using 1470 controls and 1473 HSCR patients from Southern Chinese children. The results clarified that *DOK6* rs12968648 G allele significantly increased HSCR susceptibility, in the allelic model (*p* = 0.034; *OR* = 1.12, 95%CI = 1.01~1.24) and the dominant model (*p* = 0.038; *OR* = 1.12, 95%CI = 1.01~1.25). Clinical stratification analysis showed that rs12968648 G allele was associated with increased risk of short‐segment HSCR (S‐HSCR), in the allelic model (*p* = 0.028; *OR* = 1.14, 95%CI = 1.01~1.28) and the additive model (*p* = 0.030; *OR* = 1.14, 95%CI = 1.01~1.28). *ABHD1* rs2304678 C allele had higher risk to develop total colonic aganglionosis (TCA) in the allelic model (*p* = 7.04E‐03; *OR* = 1.67, 95%CI = 1.15~2.43) and the dominant model (*p* = 4.12E‐03; *OR* = 1.93, 95%CI = 1.23~3.04). *DOK6* rs12968648 and *ABHD1* rs2304678 had significant intergenic synergistic effect according to logical regression (*p* = 0.0081; *OR* = 0.76, 95%CI = 0.63~0.93) and multifactor dimensionality reduction (MDR, *p* = 0.0045; *OR* = 1.25, 95%CI = 1.07~1.46). This study verified two susceptible variations of HSCR on *ABHD1* and *DOK6*. Their roles in HSCR should be conducted in further studies.

## 1 INTRODUCTION

Hirschsprung disease (HSCR) is a complex genetic disease related to the abnormal development of the enteric nervous system (ENS).^1^ During embryonic development, ENS in the gastrointestinal tract, from the front to the tail, undergoes the stages of survival, proliferation, migration and differentiation to form functional gut ganglion cells. HSCR is caused by the failure of ENS migration, and there is the longer ganglia‐free segment of the intestine when the failure occurs earlier. According to the length of the aganglionic tract, there are three types of HSCR, short‐segment HSCR (S‐HSCR), long‐segment HSCR (L‐HSCR) and total colonic aganglionosis (TCA), with percentages of approximately 80%, 15% and 5%, respectively.^2^, ^3^ There is an ethnic difference in the incidence of HSCR, with an average of one case per 15,000 European live births; however, the incidence of HSCR is three times higher in Asian populations (approximately 1 in 5000 live births).^4^ In addition, HSCR is divided into familial HSCR and sporadic HSCR, according to familial genetics. There are family aggregation and more sporadic cases in more than one‐fifth of HSCR patients.^5^ Mainly caused by genetic factors, the occurrence of HSCR is related to the heterogeneity of alleles.^6^ There are more than 24 HSCR‐related genes, most of which are related to ENS signalling pathways, including *RET*, *PHOX2B* and *NRG1*.^7^, ^8^, ^9^ However, the pathogenesis of HSCR cannot be fully explained by these genetic variations.

As a neuronal adaptor protein molecule, docking protein 6 (*DOK6*) is involved in multiple neurotrophic factor‐mediated neurite outgrowth and serves as a substrate for multiple tyrosine kinase receptors.^10^ In addition to being critical for neurodevelopment, *DOK6* is highly expressed in the developing nervous system. *DOK6* is reported to be involved in the *RET* signalling pathway and plays an important role in cell proliferation, survival, migration and invasion.^11^ Previous genome‐wide association (GWAS) studies on HSCR showed that rs12968648 had a moderate significance level (*p* = 1.12 × 10−4; *OR* = 1.733, 95%CI = 1.31~2.27). In addition, it also has high biological relevance.^12^ The single nucleotide polymorphism (SNP) rs12968648 in *DOK6* was replicated and has been used to calculate the risk value of HSCR.

α/β‐Hydrolase domain 1 (*ABHD1*) is the human homolog of and has the closest similarity to AlkB. AlkB family proteins are enzymes that can repair alkylated RNA and DNA through oxidative demethylation.^13^ By mediating the transcription of methionine (Met), *ABHD1* exerts influences on the synthesis of normal intestinal proteins and the proliferation and migration of intestinal stem cells (ISCs). In *ABHD1* ^(−/−)^ knockout mice, it was confirmed that the only phenotype reported relates to deficiencies in placental trophoblast lineage differentiation.^14^ The SNP rs2304678 in *ABHD1* is a missense mutation according to HaploReg database (https://pubs.broadinstitute.org/mammals/haploreg/haploreg.php), which suggests rs2304678 is likely to be functional. The SNP rs2304678 in *ABHD1* was replicated to explore the correlation of HSCR in this study.

There are uncertain risks of the candidate genes, especially the interactions of these susceptible genes. This study was designed to decide whether the genetic polymorphisms of *DOK6* (rs12968648) and *ABHD1* (rs2304678) were linked to HSCR in 1473 Chinese HSCR patients and 1470 controls. In our study, there is the first manifestation of two susceptibility genes in the epistatic association of *ABHD1* and *DOK6* with HSCR.

## 2 MATERIALS AND METHODS

### 2.1 Study subjects

This study has been authorized by the institutional review committee in Guangzhou Women and Children's Medical Center, Guangzhou, China. Participants and/or their legal custodians signed a written informed consent form before participating in this research. A total of 1473 southern Chinese HSCR patients received surgical treatment. Colonic specimens throughout the colon were intraoperatively obtained for pathological analysis and rectal biopsy, and according to the length of the segments, patients were diagnosed with L‐HSCR (294 patients), S‐HSCR (1033 patients) or TCA (82 patients; Table S1). Blood samples of 1470 people without a history of neurological diseases and HSCR were collected as control samples.

### 2.2 Single nucleotide polymorphisms genotyping and quality control

Based on previous extensive studies, *ABHD1* rs2304678 and *DOK6* rs12968648 were selected. Two SNPs were genotyped on all samples using the iPLEX Gold MassARRAY system (Sequenom). Hardy‐Weinberg equilibrium experiment was carried out to exclude SNPs (*p* < 0.05). The methods of SNP quality control included that: firstly, the patient/control was excluded from the final analysis if there was over 10% of the missing data of SNPs; secondly, all subjects with 10% of missing follow‐up calls were deleted. At last, four SNPs were retained for further analysis.

### 2.3 Subphenotype analysis and association analysis

The correlation between SNPs and disease was analysed by comparing the hazard of allele frequency (allele testing) between controls and patients, and other measurements were determined using an additive measurement of logistic regression, dominant and recessive model testing in PLINK 1.9.^15^, ^16^ The association of subphenotype stratification was analysed by comparing certain subphenotypes in the patients and controls.

### 2.4 Genetic epistasis

The genetic association was estimated using the univariate logistic regression analysis. Multifactor dimensionality reduction (MDR) was applied for the statistical analysis of gene‐gene interactions. The open‐source MDR package 2.0 was used to implement MDR analysis, and this software can be obtained online for free (http://www. Epistasis.org).^17^ Each cell in the 3 × 3 interaction table was assigned as having a low or high risk, and the genotype dimension was decreased from N to 1 through assembling these genotype combinations into two‐level variables. Permutation tests and cross‐validation (CV) were used to assess the ability of one‐dimensional genotype variable quantities to predict and classify the disease state.^18^ The threshold for differentiating genotypes from various risks was equivalent to the proportion of controls and patients in the input database. The prediction error and mean CV consistency were calculated, and the models with minimum average prediction error and maximum average CV consistency were selected, so as to decrease the biases or false results caused by the partition of the opportunity database. Under the unrelated zero hypothesis, the distribution of the mean prediction error was compared with the mean prediction error of the observation data to determine the statistical significance. The invalid hypothesis was rejected when the permutation test had the *p* value at 0.05. Logistic regression analysis is then used to measure gene‐gene interactions. Multivariate logistic regression analysis was used to adjust for latent mingle factors, including the patient's age, gender, family history of disease and ischaemic stroke (IS). The significance level of the Bonferroni correction (*p* < 0.008) was employed to carry out single‐locus analysis correction for multiple tests. Significantly statistical differences were found at *p* = 0.05 in other two‐tailed tests.

The epistasis test of logistic regression analysis (case‐control) was used to analyse the parameters of genetic interaction via PLINK 1.9.^19^, ^20^ In PLINK, a model was used based on the allele dose of 0 to 2 representing the number of risk alleles of each SNP, SNP A and SNP B, and it was matched in the layout of Y = b_0_ + b_1_ SNP A + b_2_ SNP A + b_3_ SNP A*SNP B + e. Wherein, b_1_, b_2_ and b_3_ represent the effect of the interaction between SNP A and SNP B, and the interaction testing is dependent on b3. Besides, *p* < 0.05 indicated that the difference was statistically significant.

Multifactor dimensionality reduction analysis was used to carry out the pairwise nonparametric epistasis test.^21^, ^22^ To minimize false‐positive results, these methods checked the data several times through the CV/permutation test process. To determine the statistical significance, the average prediction error of the observed data is compared with the average prediction error distribution under the null hypothesis.

## 3 RESULTS

### 3.1 Association of *DOK6* and *ABHD1* SNPs with HSCR

On *DOK6* and *ABHD1*, one SNP was chosen for replication. The method shows the selection criteria. In Table 1, there is information on two SNPs genotyped with 1473 HSCR patients and 1470 controls from South China. The association between rs2304678 in *ABHD1* and HSCR disease has not be replicated in our population (Table 1). However, the SNP rs12968648 in the *DOK6* gene was increased HSCR susceptibility (Table 1). Four genetic models are designated, namely allele, dominant, recessive and additive models to demonstrate the effective pattern for the SNP rs12968648 on *DOK6*. There is a larger effect on HSCR for the SNP rs12968648, both in the allelic model (*p* = 0.034; *OR* = 1.12, 95% CI = 1.01~1.24) and in the dominant model (*p* = 0.038; *OR* = 1.12, 95%CI = 1.01~1.25; *P* ***_adj_*** = 0.02; *OR* ***_adj_*** = 1.14, 95%CI = 1.02~1.28).

**TABLE 1:** Replication results on two selected single nucleotide polymorphisms (SNPs) in Southern Chinese population using 1470 cases and 1473 controls

CHR	SNP	*Gene*	BP	A1/A2	F_A	F_U	TEST	*p*	*P_adj_*	*OR* (95%CI)	*OR_adj_* (95%CI)
2	rs2304678	*PREB/ABHD1*	27130639	C/G	0.16	0.15	ALLELIC	0.43		1.06 (0.92~1.22)	
							DOM	0.29	0.24	1.09 (0.93~1.28)	1.11 (0.93~1.32)
							REC	0.66	0.48	0.90 (0.58~1.42)	0.84 (0.52~1.35)
							ADD	0.75	0.43	0.96 (0.77~1.21)	1.06 (0.91~1.23)
18	rs12968648	*DOK6*	69476458	G/C	0.44	0.42	ALLELIC	**0.034**		1.12 (1.01~1.24)	
							DOM	0.065	0.06	1.16 (0.99~1.36)	1.18 (0.99~1.40)
							REC	0.11	0.07	1.17 (0.97~1.41)	1.20 (0.98~1.48)
							ADD	**0.038**	**0.02**	1.12 (1.01~1.25)	1.14 (1.02~1.28)

Abbreviations: A1/A2 indicates the effect allele and reference allele to disease; BP, base pair of where the SNP is located; CHR, chromosome; F_A/F_U indicates risk allele frequency of the SNP in cases or controls; Gene.refgene: The gene where the SNP located to; SNP, single nucleotide polymorphism.

Bold values indicate *p* < 0.05 and are considered significant.

ALLELIC, DOM, REC and ADD indicate the association test following allelic genetic, dominant, recessive and additive models. The *p* value indicates the significance based on allelic association tests. *P_ad_* ***_j_***: *p* value adjusted by gender and age; the calculation of odds ratio (OR) is also based on the risk allele of each SNP. *OR_adj_*, OR adjusted by gender and age.

### 3.2 Clinical stratification of SNPs in *DOK6* and *ABHD1* with HSCR

In terms of clinical practice, different patients have different subclinical manifestations, including S‐HSCR, L‐HSCR and TCA. In this study, the association between two SNPs (including rs2304678 and rs12968648) and different clinical subtypes with HSCR was further explored. As shown in Table 2, the rs12968648 G allele significantly increased susceptibility in S‐HSCR, in the allelic model (*p* = 0.028; *OR* = 1.14, 95%CI = 1.01~1.28), the additive model (*p* = 0.030; *OR* = 1.14, 95%CI = 1.01~1.28; *P* ***_adj_*** = 0.02; *OR* ***_adj_*** = 1.16, 95%CI = 1.03~1.31) and the recessive model (*P* ***_adj_*** = 0.05; *OR* ***_adj_*** = 1.24, 95%CI = 1.00~1.55), but no significant association was found neither in L‐HSCR nor in TCA. For the rs2304678, G allele significantly increased the risk of TCA, in the allelic model (*p* = 7.04E‐03; *OR* = 1.67, 95%CI = 1.15~2.43), the dominant model (*p* = 4.12E‐03; *OR* = 1.93, 95%CI = 1.23~3.04; *P* ***_adj_* **= 0.01; *OR* ***_adj_*** = 1.96, 95%CI 0.95 = 1.19~3.25) and the additive model (*P* ***_adj_*** = 0.01; *OR* ***_adj_*** = 1.78, 95%CI = 1.15~2.77), but *p* value was not significant in S‐HSCR and L‐SHCR.

**TABLE 2:** The association results of two independent single nucleotide polymorphisms (SNPs) with different subclinical features classified by aganglionosis length, including short‐length (S‐HSCR), long‐length (L‐HSCR) and total colonic aganglionosis (TCA)

Disease status	CHR	SNP	BP	A1/A2	F_A	F_U	TEST	*p*	*P_adj_*	*OR*(95%CI)	*OR_adj_* (95%CI)
SHCSR	2	rs2304678	27130639	C/G	0.16	0.15	ALLELIC	0.37		1.07 (0.92~1.26)	
							DOM	0.31	0.18	1.10 (0.92~1.31)	1.14 (0.94~1.37)
							REC	0.98	0.83	1.01 (0.62~1.63)	0.95 (0.58~1.56)
							ADD	0.90	0.28	1.02 (0.80~1.30)	1.09 (0.93~1.28)
	18	rs12968648	69476458	G/C	0.45	0.42	ALLELIC	**0.028**		1.14 (1.01~1.28)	
							DOM	0.063	0.06	1.18 (0.99~1.40)	1.20 (1.00~1.44)
							REC	0.086	**0.05**	1.20 (0.97~1.48)	1.24 (1.00~1.55)
							ADD	**0.030**	**0.02**	1.14 (1.01~1.28)	1.16 (1.03~1.31)
L‐HSCR	2	rs2304678	27130639	C/G	0.13	0.15	ALLELIC	0.27		0.86 (0.67~1.12)	
							DOM	0.46	0.50	0.90 (0.67–1.20)	0.90 (0.66~1.22)
							REC	0.17	0.28	0.48 (0.17–1.36)	0.56 (0.19~1.61)
							ADD	0.28	0.36	0.87 (0.67–1.12)	0.88 (0.67~1.16)
	18	rs12968648	69476458	G/C	0.44	0.42	ALLELIC	0.38		1.08 (0.90~1.30)	
							DOM	0.26	0.38	1.17 (0.89–1.54)	1.14 (0.85~1.53)
							REC	0.84	0.98	1.04 (0.74–1.45)	1.00 (0.70~1.42)
							ADD	0.38	0.56	1.09 (0.90–1.30)	1.06 (0.87~1.29)
TCA	2	rs2304678	27130639	C/G	0.23	0.15	ALLELIC	**7.04E−03**		1.67 (1.15~2.43)	
							DOM	**4.12E−03**	**0.01**	1.93 (1.23~3.04)	1.96 (1.19~3.25)
							REC	0.68	0.50	1.29 (0.39~4.25)	1.69 (0.36~7.91)
							ADD	0.44	**0.01**	1.27 (0.69~2.32)	1.78 (1.15~2.77)
	18	rs12968648	69476458	G/C	0.44	0.42	ALLELIC	0.60		1.09 (0.79~1.50)	
							DOM	0.99	0.39	1.00 (0.62~1.62)	1.27 (0.73~2.20)
							REC	0.35	0.26	1.30 (0.75~2.26)	1.41 (0.77~2.56)
							ADD	0.50	0.24	1.11 (0.81~1.52)	1.24 (0.87~1.76)

Abbreviations: A1/A2 indicates the effect allele and reference allele to disease; BP, base pair of where the SNP is located; CHR, chromosome; F_A/F_U indicates risk allele frequency of the SNP in cases or controls; Gene.refgene, The gene where the SNP located to; SNP, single nucleotide polymorphism.

Bold values indicate *p* < 0.05 and are considered significant.

ALLELIC, DOM, REC and ADD indicate the association test following allelic genetic, dominant, recessive and additive models. The *p* value indicates the significance based on allelic association tests; *P_ad_* ***_j_***: *p* value adjusted by gender and age; the calculation of odds ratio (OR) is also based on the risk allele of each SNP. *OR_adj_*, OR adjusted by gender and age.

### 3.3 Intergenic SNPs show an epistatic effect on HSCR

**FIGURE 1:**
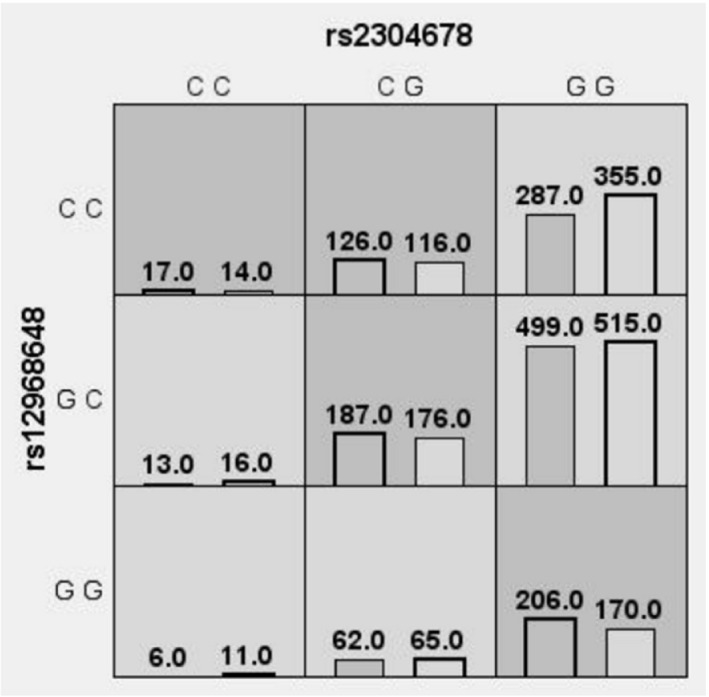
Distribution of the rs12968648 and rs2304678 genotype combinations. The rs12968648 and rs2304678 genotype combinations associated with a high risk and with a low risk for HSCR, along with the corresponding distribution of patients (left bars in cells) and of controls (right bar in cells). Dark‐shaded cells indicate a high risk of HSCR (rs12968648 CC + rs2304678 CC, rs12968648 CC + rs2304678 CG, rs12968648 GC + rs2304678 CG and rs12968648 GG + rs2304678 GG); light‐shaded cells indicate a low risk of HSCR (rs12968648 CC + rs2304678 GG, rs12968648 GC + rs2304678 CC, rs12968648 GC + rs2304678 GG, rs12968648 GG + rs2304678 CC and rs12968648 GG + rs2304678 CG)

Studies have shown that the associated SNPs increase the risk of HSCR disease by epistatic interactions. Epistatic test by logistic regression was adopted for parametric analysis using PLINK in current study. In Table 3 (left bottom), the results show that the epistatic effect between SNPs rs12968648 and rs2304678 was significantly increased in HSCR (*p* = 0.0081; *OR* = 0.76, 95%CI = 0.63~0.93). To further verify the significant epistatic effect supported by logistic regression, pairwise multifactor dimensionality reduction (MDR) analysis was applied as a statistical method to predict the epistatic effect by multiple examinations of the data. In agreement with the results by logistic regression analysis, a significant effect from synergistically epistatic interaction between rs2304678 and rs12968648 for HSCR was obtained (*p* = 0.0045; *OR* = 1.25, 95%CI = 1.07~1.46), as shown in Table 3 (right top). In Figure 1, generated by MDR analysis, there are detailed risk genotype combinations. The combinations of higher risk groups were classified. Conforming to the risk genotypes of rs12968648 (GG) and rs2304678 (GG) for single SNP associations, there is a significantly higher risk of disease by means of the chi‐square test in the combination GG‐GG. The dark‐shaded cells display a higher risk of HSCR (rs12968648 CC + rs2304678 CC, rs12968648 CC + rs2304678 CG, rs12968648 GC + rs2304678 CG and rs12968648 GG + rs2304678 GG), and the light‐shaded cells display a lower risk of HSCR (rs12968648 CC + rs2304678 GG, rs12968648 GC + rs2304678 CC, rs12968648 GC + rs2304678 GG, rs12968648 GG + rs2304678 CC and rs12968648 GG + rs2304678 CG). Overall, these results suggest the gene‐gene interaction.

**TABLE 3:** Pairwise epistatic interaction results among two variants in *ABHD1* and *DOK6* carried out by logistic regression analysis and multifactor dimensionality reduction (MDR) analysis

Gene	SNP	Method	*DOK6* rs12968648	*ABHD1* rs2304678
				Logistic regression
*DOK6*	rs12968648		NA	*OR* (CI 0.95) = 0.76 (0.63~0.93) ***p* *= *0.0081**
*PREB/ABHD1*	rs2304678	MDR	*OR* (CI 0.95) = 1.25 (1.07~1.46) ***p* = 0.0045**	NA

Abbreviations: MDR, multifactor dimensionality reduction; NA, not available; SNP, single nucleotide polymorphism; the calculation of odds ratio (OR) is also based on the risk allele of each SNP.

Bold values indicate *p* < 0.05 and are considered significant.

## 4 DISCUSSION

Hirschsprung disease is a complex congenital disease whose pathogenesis is related to several genes. An attempt was made to find extra loci associated with HSCR by designing an association study for the case‐control or trio study. In this study, two SNPs were identified on *ABHD1* and *DOK6* and related to HSCR using the largest population‐based study of HSCR with 1470 patients and 1473 controls. The *DOK6* rs12968648 G allele significantly increased HSCR susceptibility (Table 1). The in‐depth analysis of subclinical manifestations further elaborated SNP rs12968648 in *DOK6*, indicating the increased risk of S‐HSCR. In addition, the *ABHD1* rs2304678 C allele was further replicated had higher risk to develop TCA (Table 2). It is worth noting that the interaction between SNPs confirmed by logistic regression analysis and MDR analysis indicated a significant interaction between the genotypes of the SNPS of *ABHD1* rs2304678 and *DOK6* rs12968648 (Table 3).

*DOK6* is downstream of the receptor tyrosine kinase (RTK)/docking protein (DOK) family, whose proteins belong to adaptor proteins serving as substrates for various RTKs and non‐RTKs.^11^ The tropomyosin‐associated kinase (Trk) receptor family is composed of three members (TrkA, TrkB and TrkC), which are RTKs that play a key role in almost all stages of neurodevelopment, including differentiation, survival, proliferation and migration.^23^, ^24^ Wei Qili et al.^25^ showed that *DOK6* selectively binding to TrkC receptors by the domain of its phosphotyrosine‐binding (PTB) domain depended on kinase activity and that *DOK6* participated in NT‐3‐mediated neurite growth in the cortical neurons of mice as a new substrate of TrkC receptors. The in situ hybridization results showed that *DOK6* is expressed in the nervous system and is more prominent in cortical and dorsal root ganglia (DRG) neurons. Whether *DOK6* causes HSCR through this pathway remains to be proven by experiments. In addition, the correct migration of enteric neural crest cells (ENCCs) plays avital role in the development of ENS. Pachnis et al.^26^ showed that the migration of ENCCs was associated with the activation of receptor tyrosine kinases such as RETs. In the general population, the mutation of c‐RETs was associated with approximately 50% of HSCR patients.^27^ RET is a crucial signalling component of multisubunit receptors for glial cell line‐derived neurotrophic factor (*GDNF*) and other family members, such as artemin, neurturin and persephin.^28^ Signalling pathways that are mediated by the RTK *RET* have been independently identified as the key to mammalian intestinal neurogenesis.^29^ Crowder et al. showed that *DOK6* is localized in the plasma membrane through its pleckstrin homology (PH) domain and is phosphorylated upon the activation of *RET* by the mutation of multiple endocrine neoplasia type 2A (*MEN2A*) or the stimulation of *GDNF*, and *DOK6* is a substrate of the *RET* signalling pathway.^11^, ^25^, ^30^, ^31^ According to reports, *DOK6* participates in the *RET* signalling pathway and plays an important role in cell survival, proliferation, migration and invasion.^11^ It was boldly speculated that *DOK6* acted as a substrate for the *RET* signalling pathway and interfered with the normal migration of ENCCs through the RET/GDNF signalling pathway, thereby leading to HSCR. It was identified that SNP rs12968648 in *DOK6* was associated with HSCR, but further analysis showed that this SNP impacts only patients with S‐HSCR. Considering the number of samples collected, *DOK6* rs12968648 was associated with S‐HSCR, and S‐HSCR showed the highest prevalence among subtypes.

It has been reported that *ABHD1* is localized in mitochondria and particularly interacts with mitochondrial transfer RNA for Met (mt‐tRNAMet).^32^, ^33^ With a unique genome, mitochondria are expressed using an nontraditional genetic code. In addition to these genes, two mt‐rRNAs and a whole set of 22 mt‐tRNAs are contained in the mitochondrial transcriptome. The single mt‐tRNAMet not only reads the standard AUG (Met) codon but also recognizes AUA (Ile) in the process of translation initiation and elongation.^34^ As a dioxygenase, *ABHD1* oxidizes m5C34 of mt‐tRNAMet for the generation of the f5C34 modification. Multiple modifications generated in RNAs are involved in translation and may influence protein synthesis. The downregulation of *ABHD1* levels leads to a significant reduction in mitochondrial translation in vivo, indicating the crucial role of modifications induced by ABHD1 in the function of mt‐tRNAMet. *ABHD1* and a series of other modifications occur in mt‐tRNAMet, and mt‐tRNAMet mediates the incorporation of Met on codons during the course of translation initiation and elongation and identifies different codons to encode Met.^33^ As an essential amino acid, Met is crucial for the normal digestion, nutrition absorption and immune monitoring of mucosal epithelial cells and may mitigate intestinal damage by improving the proliferation of ICSs and reactivation of the Wnt/β‐catenin signalling pathway.^35^, ^36^ Increased maternal Met intake boosts the intestinal growth of newborns by promoting morphological development or upregulating the expression of genes involved in nutrient metabolism.^37^ It was hypothesized that *ABHD1* affected the normal synthesis of intestinal proteins and impeded the normal proliferation and migration of ICSs, relating to the neurodevelopment of HSCR. A significant epistatic association was shown to exist between the SNP rs12968648 in *DOK6* and the SNP rs2304678 in *ABHD1*. The analysis of subclinical manifestations further showed that the SNP rs2304678 in *ABHD1* was associated with an increased risk of TCA.

We are based on the study of the largest sample size (1740 cases and 1743 controls) of the Southern Chinese population based on the tertiary hospital centre so far. However, there are still several limitations in our research. First of all, future studies are still needed to increase the sample size for improving statistical efficiency. Secondly, the interaction of genetic environment and other exposure factors of patients (such as drug consumption) have not been emphasized. Third, the causal relation between genes and disease is not illustrated. Functional study of the gene should be conducted. Additionally, two SNPs are limited to elucidate the relationship between genes and disease. Therefore, more polymorphisms should be involved.

In summary, a systematic analysis of two candidate genes identified that the *DOK6* rs12968648 G allele significantly increased the susceptibility in HSCR, especially S‐HSCR, and the *ABHD1* rs2304678 C allele increased the risk of severe TCA. This study indicated that the risk of HSCR was increased through the genetic interaction of relevant variants between *DOK6* and *ABHD1*. This finding may determine *DOK6* and *ABHD1* as genes related to HSCR, improve the understanding of the aetiology of HSCR, but further studies are needed to verify their roles in HSCR.

## CONFLICT OF INTERESTS

The authors declare that the study was performed without any financial or commercial relations that could be interpreted as potential conflicts of interest. The authors have no conflicts of interest to declare.

## AUTHOR CONTRIBUTIONS

**Chaoting Lan:** Data curation (equal); Investigation (equal); Writing‐original draft (lead). **Yuxin Wu:** Data curation (equal); Investigation (equal). **Ning Wang:** Data curation (equal); Investigation (equal). **Yun Luo:** Investigation (equal). **Jinglu Zhao:** Investigation (equal). **Yi Zheng:** Funding acquisition (equal); Investigation (equal). **Yan Zhang:** Funding acquisition (equal); Investigation (equal). **Lihua Huang:** Funding acquisition (equal); Investigation (equal). **Yun Zhu:** Investigation (equal). **Lifeng Lu:** Investigation (equal). **Wei Zhong:** Funding acquisition (equal); Investigation (equal). **Jixiao Zeng:** Data curation (equal); Methodology (equal). **Huimin Xia:** Data curation (equal); Funding acquisition (lead); Methodology (lead); Writing‐review & editing (lead).

## Supporting information

Table S1

## Data Availability

All data included in this study are available upon request by contact with the corresponding author.
