# Supplementary material for: Association between ABHD1 and DOK6 polymorphisms and susceptibility to Hirschsprung disease in Southern Chinese children
Source: J Cell Mol Med. 2021 Sep 20;25(20):9609–16. doi: 10.1111/jcmm.16905 (PMC8505836; doi:10.1111/jcmm.16905)
Supplement: Supplementary file 1 — Table S1 [file JCMM-25-9609-s001.docx]

| **Supplemental Table 1. Frequency distribution of selected characteristics in the cases and the controls** | | | | | |
| --- | --- | --- | --- | --- | --- |
| **Variables** | **Cases**  **(n=1470)** | | **Controls**  **(n=1473)** | | ***P*** |
|  | No. | % | No. | % |  |
| Age (Months) |  |  |  |  |  |
| Mean ± SD | 8.37±20.50 | | 18.61±19.75 | | **＜0.001**^b^ |
| ≤2 | 725 | 49.32 | 458 | 31.09 |  |
| >2 | 745 | 50.68 | 1015 | 68.91 | **＜0.001**^a^ |
| Gender (Female/Male) |  |  |  |  |  |
| Female | 240 | 16.33 | 458 | 65.65 |  |
| Male | 1230 | 83.67 | 1015 | 34.35 | **＜0.001**^a^ |
| Clinical manifestation |  |  |  |  |  |
| SHSCR | 1033 | 70.27 | / | / |  |
| LHSCR | 294 | 20.00 | / | / |  |
| TCA | 82 | 5.58 | / | / |  |
| Total intestine | 3 | 0.20 | / | / |  |
| NA | 58 | 0.40 | / | / |  |
| SD, standard deviation; NA, not available.  ^a^ Two-sided χ^2^ test for distributions between HSCR cases and controls.  ^b^ Two-sided t test for distributions between HSCR cases and controls. | | | | | |
